# Extracorporeal immune therapy with immobilized agonistic anti-Fas antibodies leads to transient reduction of circulating neutrophil numbers and limits tissue damage after hemorrhagic shock/resuscitation in a porcine model

**DOI:** 10.1186/1476-9255-7-18

**Published:** 2010-04-20

**Authors:** Tim T Lögters, Jens Altrichter, Adnana Paunel-Görgülü, Martin Sager, Ingo Witte, Annina Ott, Sarah Sadek, Jessica Baltes, José Bitu-Moreno, Alberto Schek, Wolfram Müller, Teresa Jeri, Joachim Windolf, Martin Scholz

**Affiliations:** 1 Department of Trauma and Hand Surgery, University Hospital, Düsseldorf, Germany; 2 Department of Thoracic and Cardiovascular Surgery, University Hospital, Frankfurt am Main, Germany; 3 Department of Vascular Surgery, Faculdade Medicina Marilia (FAMEMA), Marilia, Brasil; 4 Pathology Group Starnberg, Starnberg, Germany

## Abstract

**Background:**

Hemorrhagic shock/resuscitation is associated with aberrant neutrophil activation and organ failure. This experimental porcine study was done to evaluate the effects of Fas-directed extracorporeal immune therapy with a leukocyte inhibition module (LIM) on hemodynamics, neutrophil tissue infiltration, and tissue damage after hemorrhagic shock/resuscitation.

**Methods:**

In a prospective controlled double-armed animal trial 24 Munich Mini Pigs (30.3 ± 3.3 kg) were rapidly haemorrhaged to reach a mean arterial pressure (MAP) of 35 ± 5 mmHg, maintained hypotensive for 45 minutes, and then were resuscitated with Ringer' solution to baseline MAP. With beginning of resuscitation 12 pigs underwent extracorporeal immune therapy for 3 hours (LIM group) and 12 pigs were resuscitated according to standard medical care (SMC). Haemodynamics, haematologic, metabolic, and organ specific damage parameters were monitored. Neutrophil infiltration was analyzed histologically after 48 and 72 hours. Lipid peroxidation and apoptosis were specifically determined in lung, bowel, and liver.

**Results:**

In the LIM group, neutrophil counts were reduced versus SMC during extracorporeal immune therapy. After 72 hours, the haemodynamic parameters MAP and cardiac output (CO) were significantly better in the LIM group. Histological analyses showed reduction of shock-related neutrophil tissue infiltration in the LIM group, especially in the lungs. Lower amounts of apoptotic cells and lipid peroxidation were found in organs after LIM treatment.

**Conclusions:**

Transient Fas-directed extracorporeal immune therapy may protect from posthemorrhagic neutrophil tissue infiltration and tissue damage.

## Background

Hemorrhagic shock is a leading cause of complications and death in combat casualties and civilian trauma [1]. It has been shown to cause systemic inflammatory response syndrome (SIRS), multiple organ dysfunction syndrome (MODS), and multiple organ failure (MOF) [2]. Despite intensive investigations, the pathophysiology of posthemorrhagic multiple organ failure remains incompletely understood. Recently, it has been reported that neutrophils recruited by mitochondrial products (formyl peptides and mitochondrial DNA) released from damaged tissues and cells are responsible for the inflammation seen in SIRS [3]. However, tissue infiltration with activated polymorphonuclear neutrophils is associated with collateral tissue damage elicited by excessive amounts of neutrophil-derived proteases and oxygen radicals which may affect all major organs and largely contribute to MODS [4-17].

One major reason for the collateral damage mediated by hyperactivated neutrophils is the prolonged neutrophil survival time in conjunction with resistance against apoptosis [18]. There is increasing evidence that prolonged neutrophil survival is due to reduced susceptibility to proapoptotic mediators as a result of proinflammatory cytokines [19] and cytokines [20]. Moreover, intracellular inhibitors of apoptosis proteins (IAPs) are important regulators of neutrophil survival time under inflammatory conditions [21]. Unfortunately, the role of modified neutrophil susceptibility against proapoptotic signaling in the posttraumatic/posthemorrhagic situation and its potential for therapeutic targeting is largely unknown.

Recently, we developed an extracorporeal immune therapy approach to inactivate circulating neutrophils by targeting neutrophil Fas [22-25]. It is known that adequate cross-linking of Fas (APO-1, CD95) on the neutrophil surface membrane stimulates proapoptotic signaling pathways [26,27] but probably may also lead to cellular changes independent from apoptosis [28]. In this regard, we could show earlier that neutrophils rapidly become inactive following contact with membrane bound FasL [29] or with immobilized agonistic anti-Fas IgM antibody [24]. Moreover, evidence has been obtained that the transient contact of technetium-labelled neutrophils with immobilized anti-Fas IgM leads to their rapid sequestration in the spleen [22]. This proposed mechanism might efficiently reduce the number of preapoptotic circulating neutrophils within the circulation. In addition, we recently showed that apoptosis resistance of hyperactivated neutrophils from patients with major trauma may be overcome by agonistic Fas stimulation [30] which may also lead to a shorter life time of activated circulating neutrophils.

This experimental study was done to find out whether neutrophil Fas-directed extracorporeal immune therapy may limit posthemorrhagic inflammation and MODS. Therefore, an extracorporeal mini circuit was developed for the use in a porcine hemorrhagic shock model. As the functional unit, a down-scaled adaptation of the anti-Fas containing leukocyte inhibition module (LIM) as it was used previously for the integration in heart-lung machines [24] was connected to the circuit. The module allows Fas specific inactivation of circulating neutrophils at a flow of 300 ml/min. At this flow neutrophils adhere to and roll over biofunctionally modified three dimensional polyurethane surfaces that carry covalently immobilized anti-Fas (anti-CD95) monoclonal IgM antibodies. Upon contact with the biofunctional surface, inactivated neutrophils rapidly lose their ability to adhere and to migrate towards chemotactic signals [12,29]. Consequently, neutrophils detach from the artificial surface and may be efficiently cleared from the blood probably by phagocytic engulfment [31] and degradation in the spleen [22].

To define whether this specific extracorporeal immune therapy is superior over standard medical care, one group of animals was hemorrhaged/resuscitated without any further treatment whereas the verum group underwent posthemorrhagic extracorporeal immune therapy with the mini-circuit.

## Methods

### Animals and groups

The animal experiments were performed according to the National Institutes of Health Guidelines for the use of experimental animals. This study was approved by the regional government of Düsseldorf and supervised by the animal health officer of the University of Düsseldorf. Twenty-four pigs (Munich mini pigs; 30.3 ± 3.3 kg) were allocated to 2 groups (each n = 12). All animals were fasted 24 hours before surgery and only received water ad libitum. For histological control samples five additional untreated healthy animals were sacrificed.

### Premedication and anesthesia

The animals were premedicated with ketamine and azaperon. Pigs were anesthetized with analgosedation (Thiopental), relaxed, and intubated endotracheally. Ventilation was performed with Isoflurane (1%) and nitrous oxide:oxygen (3:1) mixture with a tidal volume adjusted to maintain PaCO_2 _values between 36 and 44 Torr [4.8 and 5.9 kPa] and PaO_2 _between 100 and 150 Torr [13.3 and 20 kPa].

### Surgical preparation

All invasive procedures were accomplished using aseptic technique. Several catheters were inserted for hemodynamic monitoring, blood sampling and connection of the circuits for LIM. A median cut at the ventral neck was accomplished to allow insertion of a 5-Fr catheter into the left carotid artery for continuous arterial pressure monitoring. An 8-Fr Sheldon catheter was placed into the left external jugular vein. This catheter was used for controlled hemorrhage, extracorporeal circulation, and intermittent blood sampling. In addition an 8-Fr introducer sheath was placed into the right external jugular vein followed by a Swan-Ganz catheter (Edwards Lifesciences, Irvine, California, USA) insertion. After verifying proper calibration of arterial and Swan-Ganz-catheter all catheters were fixed subcutaneously.

### Extracorporeal Fas-targeted immune therapy with the Leukocyte inhibition module (LIM)

**Figure 1:**
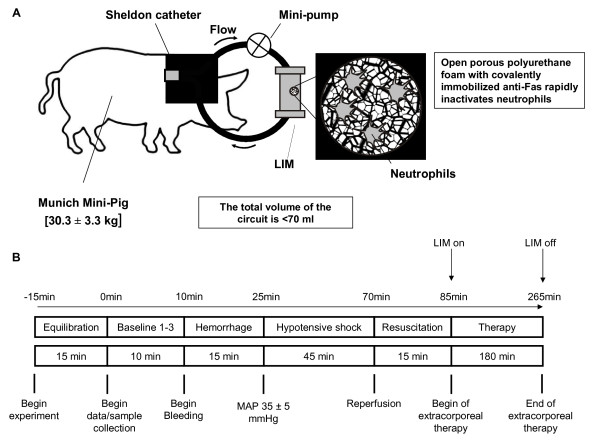
**Scheme (A) of the Fas-directed extracorporeal immune therapy (LIM) in the porcine model and (B) schematic depiction of experimental procedures over time**.

The extracorporeal immune therapy circuit (Figure 1) consists of a Sheldon catheter, a tubing set, and a functional unit with a total volume of 70 ml housing an open porous polyurethane foam with specific 3-dimensional characteristics that allows blood flow of 300 ml/min. The foam is coated with anti-Fas (CD95/APO-1) directed agonistic antibodies (clone CH11). The circuit was primed with 70 ml Ringer' solution. After anticoagulation by means of systemic administration of 200 IU/kg heparin (Liquemin; Roche, Grenzach-Wyhlen, Germany) the housing was connected with both lines to the Sheldon catheter (Fig. 1A). To rule out a possible bias, pigs undergoing hemorrhagic shock/resuscitation without extracorporeal immune therapy (standard medical care; SMC) received the same amounts of heparin.

### Experimental protocol

All animals were allowed to equilibrate for 15 minutes before baseline measurements (time point 0; Figure 1B). After two additional baseline measurements within 10 minutes, each animal was hemorrhaged rapidly through the Sheldon catheter over 15 minutes in order to reach a mean arterial pressure (MAP) of 35 ± 5 mmHg. Average volume of withdrawn blood was 586 ± 22 ml (SMC: 555 ± 34 ml; LIM: 616 ± 26 ml, n.s.). All animals were kept hypotensive for the next 30 minutes at an MAP of 35 ± 5 mmHg and for further 15 minutes at 40 ± 5 mmHg.

Subsequently, resuscitation was carried out by transfusion of 961 ± 28 ml crystalloid (Ringer') solution back to about 90% of the baseline MAP level (SMC: 916 ± 50 ml; LIM: 1005 ± 18 ml, n.s.). Fifteen minutes after resuscitation extracorporeal circuits were connected to the Sheldon catheter and extracorporeal circulation was initiated with a flow rate of 300 ml/min (LIM group, n = 12). After 3 hours the circuit was flushed with Ringer's solution and disconnected. All animals were then allowed to recover and observed for 48 hours (n = 12, 6 of each group) or 72 hours (n = 12, 6 of each group). Then animals underwent anesthesia, intubation and ventilation again. Catheters were reconnected and after a steady-state stabilization period of 30 minutes hemodynamic parameters were examined for 15 minutes. Finally, pigs were sacrificed and autopsy was performed.

### Hemodynamics

During anesthesia following hemodynamic variables were continuously measured with Swan-Ganz and arterial catheter: mean arterial pressure (MAP), heart rate (HR), cardiac output (CO), central venous pressure (CVP), pulmonary capillary wedge pressure (PCWP), mean pulmonary arterial pressure (MPAP), and central venous oxygen saturation (svO2). Blood gas samples were collected every 10 minutes throughout the experimental procedure and measured with a blood gas analysis system (ABL800 Flex, Radiometer GmbH, Willich, Germany). From beginning of baseline measurements venous blood samples were collected at time points 10, 25, 70, 85, 95, 115, 145, 205, 265 minutes as well 12, 24, 48, 72 h after surgery and were analyzed with standardized methods of clinical chemistry. Red blood count, leukocyte count and differential, erythrocyte parameters and platelets were analyzed from EDTA blood (scil animal care company GmbH, Viernheim, Germany).

### Histology and staining procedures

All animals included in this study as well as five healthy control animals without any treatment have been euthanised in order to harvest organs for histological evaluation. Tissue samples were fixed in 4% formaldehyde and embedded in paraffin according to standard procedures. Sections (5 μm) were stained with hematoxylin-eosin for pathological examination. In addition, chloracetatesterase staining was performed for specific detection and quantification of tissue infiltration by neutrophils. Neutrophils were counted in a blinded and standardized fashion by microscopy (Axiovert 40, Zeiss, Jena, Germany). Briefly, an ocular micrometer (x10) was used to count neutrophils in 10 different high power fields (HPF) of each section. Mean values from each organ and animal were allocated to predefined ranges of countings/0.09 mm^2 ^(0-5, 6-10, 11-20, 21-50, 51-100, 101-500).

### Quantification of apoptotic cells in tissue sections by TUNEL - Assay

For histological evaluation of apoptotic cells in the porcine tissues, tissue samples of lung, liver, and bowel were frozen directly after removal in liquid nitrogen and stored at -80°C before further utilization. For Tdt-mediated dUTP Nick-End Labeling (TUNEL)-Assay, samples were first embedded in paraffin and 5 μm - sections were prepared according to standard protocols. All following steps were done according to instructions of DeadEnd™ Fluorometric TUNEL System kit (Promega GmbH, Mannheim, Germany). Microscopic examination of DAPI (4'-6-Diamidin-2'-phenylindol-dihydrochlorid) stained nuclei and apoptotic domains was carried out with a fluorescence microscope (Axioskop 40, Zeiss, Jena, Germany) in 400 fold magnification. Different visual fields were selected for each tissue type to count up to 1000 DAPI positive cells. The percentage of apoptotic cells was calculated as the number of TUNEL positive cells from all DAPI positive cells counted. As a positive control for the staining procedure some slides were incubated with DNase before TUNEL staining, resulting in 100% TUNEL positive cells in each field.

### Polymerase chain reaction

Total RNA from tissue was extracted using TRI REAGENT (Sigma, Munich, Germany) according to the manufacturer's instructions. 10 μl of total RNA was reverse transcribed using oligo (dT) 15 primer (Sigma, Munich, Germany), employing Omniscript Reverse Transcriptase (Qiagen, Hilden, Germany) and following the manufacturer's instructions. PCR was carried out using gene specific primer sequences for heme oxygenase-1 (HO-1; pHO-1-R: 5'-CGTAGCGCTTGGTGGCCTGCG-3'; -F: 5'-CAGCCCAACAGCATGCCCCAG-3', Genosys-Sigma, Munich, Germany). Primers for glyceraldehyde 3-phosphate dehydrogenase (GAPDH) (hGAPDH-R: 5'-GAAGTCAGAGGAGACCACCA-3'; -F: 5'-CACCACCATGGAGAAGGCTG-3', Genosys-Sigma, Munich, Germany) were used as controls. 2.5 μl of cDNA were amplified using Taq PCR Core Kit (Qiagen, Hilden, Germany) and products were separated on 1.8% agarose gel and visualized under UV after Sybr Gold (Invitrogen, Karlsruhe, Germany) staining.

### Western blot analysis

Tissue samples were suspended in RIPA buffer (1% Nonidet-P40 (NP40), 0.5 mM sodium deoxycholate, 0.1% sodium dodecyl sulfate (SDS) in PBS) supplemented with the Complete Protease Inhibitor Cocktail (Roche, Mannheim, Germany). Samples were sonicated and incubated at 4°C for 15 min. After centrifugation at 8,000 × g for 10 min and 4°C, protein concentration was assayed using the Dc Protein Assay kit from Bio-Rad. Protein (30 μg/sample) was separated on SDS-polyacrylamide gel electrophoresis and transferred to nitrocellulose membrane. Membranes were saturated in Tris-buffered saline (TBS) containing 0.1% Tween-20 and 5% w/v nonfat dry milk (blocking buffer) for 60 min at room temperature and then incubated with mouse HO-1 monoclonal primary antibodies specific against pig HO-1 (Stressgen, Victoria, Canada) diluted in TBS containing 0.1% Tween-20 and 5% w/v nonfat dry milk. After three washes in TBS containing 0.1% Tween-20, the membranes were incubated for 60 min at room temperature with the horseradish peroxidase-labelled polyclonal goat anti-mouse secondary antibody for HO-1 (Dako Cytomation, Glostrup, Denmark), diluted 1:1,000 in TBS, 0.1% Tween-20 and washed as described above. Bands were visualized by the enhanced chemiluminescence method (SuperSignal West pico Chemiluminescent Substrate, Pierce, Bonn, Germany). Equal loading of gels was confirmed both by Ponceau S staining of membranes and by re-incubation of the filters with a polyclonal antibody for beta-Actin (Santa Cruz, Heidelberg, Germany). The amount of specific protein was quantified by densitometry (Quantity One, Bio-Rad, Munich, Germany).

### Lipid peroxidation assay

The determination of lipid peroxidation in tissue homogenates was done by quantification of thiobarbituric acid reactive substances (TBARS; Cayman Chemical Company, Ann Arbor, MI). Lipid peroxides, derived from polyunsaturated fatty acids, are unstable and decompose to form a complex series of compounds, which include reactive carbonyl compounds, such as malondialdehyde (MDA). The assay is based on the reaction of MDA with thiobarbituric acid (TBA) which is added to the sample. MDA-TBA adducts formed by the reaction of MDA and TBA under high temperature (90-100°C) and acidic conditions is measured colorimetrically at 530-540 nm (Victor 3, Perkin Elmer). Briefly, 25 mg of frozen tissue (-80°C) were mixed with RIPA buffer (1% Nonidet-P40 (NP40), 0.5 mM sodium deoxycholate, 0.1% sodium dodecyl sulfate (SDS) in PBS) with protease inhibitors (Complete Mini, Roche). The mixture was homogenized with a pestle and sonicated (Ultrasonic processor UP50H, Hielscher) for 15 seconds on ice. The tubes were then centrifuged at 1600 × g for 10 minutes at 4°C. The supernatant was used for protein concentration analysis (Dc Protein Assay, Biorad), standarized at 1 mg protein/ml solution and utilized for TBARS-assay immediately. The assay was done in duplicates in 96 well plates. Data were compared with standards provided by the manufacturer. The obtained MDA values were calculated using the formula provided by the manufacturer. The dynamic range of the kit is 0-50 μM MDA equivalents.

### Statistical analysis

Statistical analysis was carried out using the SAS/Stat for Windows software (SAS Institute, Inc, Cary, NC, version 8) and SPSS (SPSS, Inc, Chicago, IL, version 15). Non-parametric tests of the raw data were used to analyze specific inter-group and over-time differences. Data was considered to be statistically significant at p < 0.05. Wilcoxon two-sample test was used for specific inter-group (LIM versus SMC groups) difference and Wilcoxon paired test for over time differences (time point versus start value).

## Results

### Effects of LIM on leukocyte counts

**Figure 2:**
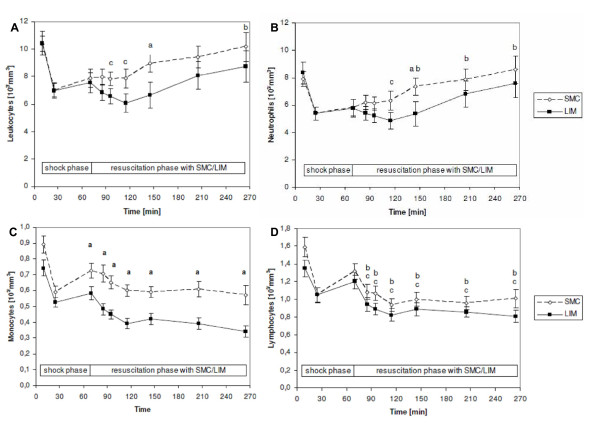
**Time kinetics of leukocyte (A), neutrophil (B), monocytes (C), and lymphocytes (D) counts for the SMC group and LIM group; n = 12 per group**. Mean ± SEM. ^a ^- Statistically significant (p < 0.05) between SMC and LIM group. ^b ^- Statistically significant (p < 0.05) difference compared with end of shock value (70 minutes) in SMC group. ^c^-Statistically significant (p < 0.05) difference compared with end of shock value (70 minutes) in LIM group.

Time kinetics of leukocyte counts was determined throughout the entire experiments (Figure 2). As shown in Figure 2A, after beginning of resuscitation with LIM leukocyte counts were found to be depressed until the end of extracorporeal immune therapy in the LIM group compared with SMC. This was due to the depression of neutrophil numbers (Figure 2B) and monocyte numbers (Figure 2C), whereas lymphocyte numbers were not significantly modified (Figure 2D). Three hours after reperfusion, neutrophil counts increased in both groups. Furthermore, 72 hours after beginning of resuscitation neutrophil counts were significantly reduced in the LIM group compared to SMC (p < 0.05). However, 24 and 48 hours after beginning of resuscitation no intergroup differences were evident for neutrophil counts (data not shown).

### Effects of LIM on hemodynamics

**Figure 3:**
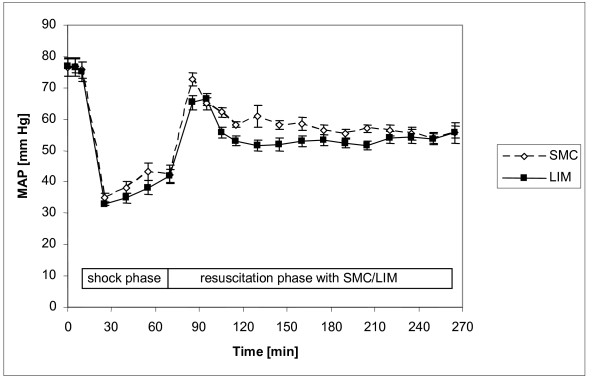
**Time kinetics of mean arterial pressure (MAP) in the shock and resuscitation phase for the LIM group and the SMC group; n = 12 per group; Mean ± SEM**. Mean arterial pressure is expressed as mmHg.

MAP in both groups was equivalent at baseline (SMC: 75.7 ± 2.57 mmHg; LIM: 75.2 ± 3.11 mmHg) and decreased in a similar pattern during hemorrhage (Figure 3). During resuscitation MAP reached 89% of the baseline levels. However, it was found to be significantly (p < 0.05) decreased in the post resuscitation period in both groups (Figure 3, Table 1). After 72 h MAP values were significantly higher in the LIM group compared with SMC (p < 0.05, Table 1). Heart rate (HR) for both groups was slightly different at baseline (SMC: 86.7 ± 3.41 beats/min; LIM: 96.2 ± 4.37 beats/min). As expected, HR increased during hemorrhage until begin of resuscitation (SMC: 128.6 ± 10.7; LIM: 164.9 ± 7.52 beats/min). HR remained increased during the post resuscitation period compared to baseline levels (data not shown). In contrast to the values for the SMC group, values for the LIM group were below baseline at 72 h (Table 1). Within the first 48 hours after resuscitation no significant improvement in hemodynamic variables (MAP, HR, CO, CVP, svO_2_, PCWP, MPAP) was observed in the LIM group. However, after 72 hours MAP and CO were significantly (p < 0.05) higher in the LIM group compared to the SMC group (Table 1). SvO_2 _was 63.1 ± 5.77% for the LIM group and 49.1 ± 3.7% for SMC (p = 0.0625).

**Table 1:** Time kinetics of hemodynamic parameters

	0 h		48 h		72 h	
	**SMC**	**LIM**	**SMC**	**LIM**	**SMC**	**LIM**

MAP [mmHg]	75.7 ± 2.57	75.2 ± 3.11	44.9 ± 2.64^*a*^	40.3 ± 4.86^*a*^	43.8 ± 2.63^*a*^	52.9 ± 2.54^*ab*^
HR [beats/min]	86.7 ± 3.41	96.2 ± 4.37	91.9 ± 6.59	105.9 ± 6.63	95.6 ± 9.77	90.0 ± 5.00
CO [l/min]	3.0 ± 0.13	3.1 ± 0.12	2.3 ± 0.23	2.3 ± 0.30	2.2 ± 0.08^*a*^	3.1 ± 0.24^*b*^
CVP [mmHg]	3.3 ± 0.70	3.8 ± 0.55	1.1 ± 0.69	5.8 ± 2.19^*b*^	3.4 ± 1.70	4.8 ± 1.24
svO_2 _[%]	86.9 ± 0.95	82.9 ± 2.69	56.0 ± 2.47^*a*^	57.7 ± 6.44^*a*^	49.1 ± 3.70^*a*^	63.1 ± 5.77
PCWP [mmHg]	7.3 ± 1.23	8.2 ± 0.57	3.8 ± 0.88	5.2 ± 0.89	5.9 ± 1.43	5.9 ± 1.12
MPAP [mmHg]	14.8 ± 1.22	17.8 ± 1.49	7.3 ± 1.01^*a*^	10.9 ± 1.10^*ab*^	12.2 ± 2.10^*a*^	13.7 ± 1.05^*a*^

MAP: mean arterial pressure, HR: heart rate; CO: cardiac output; CVP: central venous pressure; svO_2_: central venous oxygen saturation; PCWP: pulmonary capillary wedge pressure; MPAP: mean pulmonary arterial pressure; n = 12 per group at time point 0; at 48 h and 72 h, n = 6; Mean ± SEM. *a*-statistically significant (p < 0.05) over-time; *b*-statistically significant (p < 0.05) between SMC and LIM group.

### Ischemia and tissue damage parameters

Transaminases (AST, ALT), creatine phosphokinase (CK), CK-MB, Troponin T, and lactate significantly (p < 0.05) increased over time in both groups (Table 2). In conjunction with the increase in lactate, base excess (BE) significantly decreased over time. At 24, 48, and 72 hours lactate values were slightly lower in the LIM group. After 72 hours lactate values were at pre shock level in both groups. CK values were significantly lower 72 hours after shock in the LIM-treated animals (1431 ± 305 U/l) compared with the SMC group (2337 ± 232 U/l).

**Table 2:** Time kinetics of metabolic and organ specific parameters

	0 h		End shock		24 h		48 h		72 h	
	**SMC**	**LIM**	**SMC**	**LIM**	**SMC**	**LIM**	**SMC**	**LIM**	**SMC**	**LIM**

Lactate	3.3 ± 0.26	3.4 ± 0.38	3.4 ± 0.28	4.0 ± 0.43^*a*^	n.d.	n.d.	2.1 ± 0.39	2.4 ± 0.78	2.3 ± 0.22^*a*^	1.6 ± 0.31^*a*^
BE	3.1 ± 0.58	5.0 ± 0.57^*b*^	1.4 ± 0.91	1.4 ± 0.71^*a*^	n.d.	n.d.	4.3 ± 0.58	4.8 ± 1.59	5.2 ± 0.92	6.1 ± 1.05
Creatinine [1.1-1.8]	1.0 ± 0.03	0.9 ± 0.05^*b*^	1.0 ± 0.05	0.9 ± 0.06	1.2 ± 0.07^*a*^	1.2 ± 0.16	0.9 ± 0.09	1.1 ± 0.18	1.1 ± 0.06	0.8 ± 0.04^*b*^
AST [23-54]	56 ± 6.8	40 ± 2.8	37 ± 4^*a*^	31 ± 2.91	912 ± 193^*a*^	1853 ± 572^*a*^	378 ± 120^*a*^	854 ± 515^*a*^	62 ± 6.8	64 ± 9.7
ALT [50-90]	60 ± 5.68	51.1 ± 3.0	31 ± 3.3^*a*^	26 ± 1.28^*a*^	203 ± 25.3^*a*^	258 ± 33.8^*a*^	178 ± 19.2^*a*^	213 ± 30.0^*a*^	108 ± 5.84^*a*^	123 ± 16.5^*a*^
CK [251-810]	1643 ± 220	1183 ± 87	982 ± 134^*a*^	716 ± 56^*a*^	58420 ± 9767^*a*^	77653 ± 14960^*a*^	15851 ± 4185^*a*^	29439 ± 15529^*a*^	2338 ± 233	1431 ± 305^*b*^
CK-MB	180 ± 20	151 ± 6	95 ± 13^*a*^	97 ± 10^*a*^	767 ± 84^*a*^	969 ± 144^*a*^	294 ± 33	467 ± 152^*a*^	156 ± 12^*a*^	134 ± 31
Troponin T [< 0.05]	0.03 ± 0.01	0.02 ± 0.003	0.04 ± 0.01	0.04 ± 0.01^*a*^	0.08 ± 0.03	0.15 ± 0.05^*a*^	0.02 ± 0.004	0.04 ± 0.021	0.02 ± 0.006	0.01 ± 0.00

Reference Ranges in []. Lactate [mmol/l]; BE [mmol/l]: base excess; creatinine [mg/dl]; AST [U/l]: aspartate aminotransaminase; ALT [U/l]: alanine aminotransaminase; CK [U/l]: creatine phosphokinase; CK-MB [U/l]: „MB"-type isoenzyme of creatine phosphokinase; Troponin T [ng/ml]; n = 12 per group, 72 h n = 6; Mean ± SEM; *a*-statistically significant (p < 0.05) over-time; *b*-statistically significant (p < 0.05) between SMC and LIM group. n.d. = not determined.

### Neutrophil tissue infiltration

**Figure 4:**
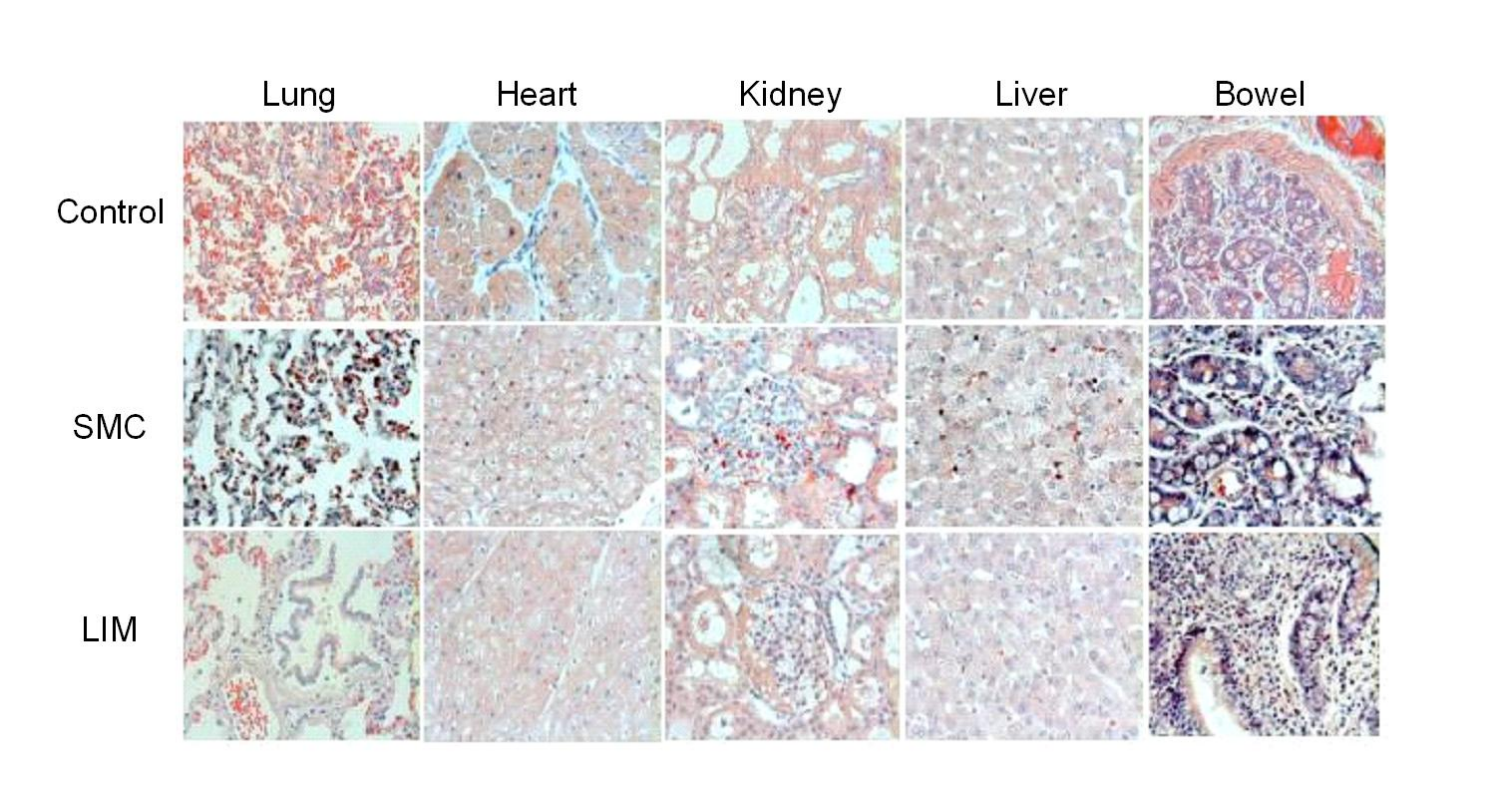
**Chloracetatesterase staining of paraffin sections from heart, lung, liver, kidney, and bowel**. Representative tissue samples for untreated healthy control pigs, pigs undergoing hemorrhage/resuscitation (SMC), and pigs undergoing hemorrhage/resuscitation with treatment (LIM). Except for control animals, organs were harvested 48 h after shock.

Representative tissue sections of lung, heart, liver, kidney, and bowel are depicted in Figure 4. Histopathological evaluation did not reveal tissue damage. However, counting of CHE positive cells/HPF revealed increase of neutrophil numbers in the tissues. All SMC animals exhibited neutrophil infiltration of the lungs versus control (SMC range: 101-500, n = 12; control range: 6-10, n = 5). Animals undergoing LIM treatment exhibited only a weak infiltration (11-20, n = 9; 21-50, n = 3). The LIM-mediated limitation of neutrophil infiltration was also found in heart (left ventricle), liver, kidneys (glomeruli), and bowel. However, the differences between SMC and LIM groups were less evident than in the lung.

### HO-1 expression, lipid peroxidation, and apoptosis

**Figure 5:**
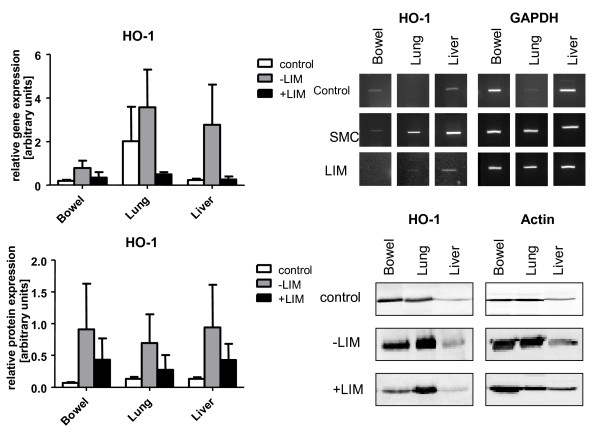
**Heme oxygenase-1 (HO-1) gene expression (A), and HO-1 protein expression (B) in control (white bars), SMC (grey bars), and LIM (black bars) animals**.

**Figure 6:**
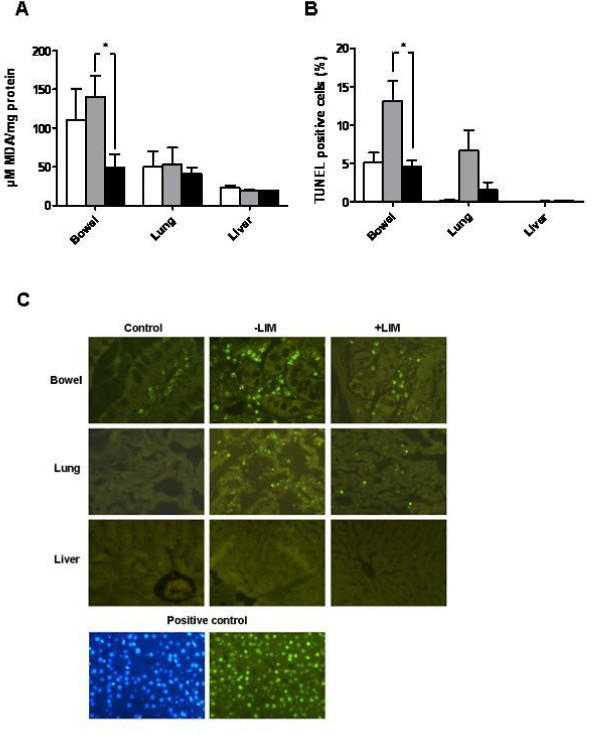
**Lipid peroxidation (A) and apoptosis (B) in bowel, lung, and liver as determined by means of malondialdehyde (MDA) assay and Tdt-mediated dUTP Nick-End Labeling (TUNEL), respectively**. Data is shown for control (white bars), SMC (grey bars), and LIM (black bars) animals. *Statistically significant (p < 0.05) difference. Positive controls indicate staining with 4'-6-Diamidin-2'-phenylindol-dihydrochlorid (DAPI; left) and TUNEL (right) after incubation of tissue with DNase.

HO-1 gene and protein expression as a counter-regulation mechanism of oxidative stress was found to be induced in bowels, lungs, and livers in animals that underwent hemorrhagic shock/resuscitation compared to control animals that did not undergo hemorrhagic shock (Figure 5). Both HO-1 gene (Figure 5A) and protein (Figure 5B) expression was lower in the LIM group as compared with SMC. In addition, MDA values that indicate lipid peroxidation and thus tissue damage were significantly lower in the bowels and slightly lower in the lungs of animals in the LIM group compared with the SMC group after shock (Figure 6A). Lipid peroxidation was not found in the livers of animals of either group when compared with control animals.

The putative contribution of apoptosis within bowels, lungs, and livers was studied by TUNEL staining. The numbers of TUNEL positive cells as the percentage from DAPI positive cells were calculated. Results are depicted as relative countings (Figure 6B) and qualitatively as microphotographs (Figure 6C). Apoptosis was lower in the lamina propria of the bowels (p < 0.05) and in the lungs (not significant) of animals in the LIM group compared with the SMC group. No Apoptosis was found by TUNEL staining in the liver.

## Discussion

In our porcine hemorrhagic shock/resuscitation model we observed impaired hemodynamics, neutrophil tissue infiltration, lipid peroxidation in the bowel, lung, and liver during an observation period of 72 hours Extracorporeal immune therapy targeting neutrophil Fas ameliorated shock-related pathophysiology. The ability of the mouse-anti-human agonistic anti-Fas IgM used in this study to induce porcine neutrophil apoptosis and to impair the effector functions was shown in earlier studies [22,25]. In previous experiments and in experiments that were done to establish this model, mini circuits without antibody coating were run to exclude effects mediated by the circuit itself. In these tests hemodynamics and leukocyte counts were similar to the SMC group. However, in the current study we may not totally exclude LIM effects that are not dependent on Fas activation on neutrophils.

Our working hypothesis was that posthemorrhagic targeting of circulating neutrophil Fas may rapidly impair neutrophil effector functions and thus may prevent their prolonged hyperactivation and neutrophil-mediated tissue damage. We previously found that binding of neutrophils to membrane-bound but not soluble FasL inactivated neutrophils within minutes even before signs of apoptosis were detectable [29], leading us to the assumption that immobilized agonistic anti-Fas may be used to therapeutically limit hyperactivation of neutrophils. In addition, functionalized biocompatible surfaces with agonistic anti-Fas in extracorporeal immune therapy may be more suitable than systemic application of anti-Fas because the latter approach has been shown to have severe side effects such as liver toxicity and pulmonary fibrosis [32,33].

Therefore, in order to effectively inactivate neutrophils in an early phase of posthemorrhagic immune deregulation, an extracorporeal circuit with a neutrophil inhibition module (LIM) on the functional basis of immobilized agonistic anti-Fas IgM was used in a porcine hemorrhagic shock/resuscitation model. The proof of concept of such an approach had been previously shown in patients undergoing cardiac surgery [24,25].

In this study, the efficacy of LIM has been shown by the relative reduction of neutrophil counts during the treatment phase. Histopathological analyses of post hemorrhagic organs clearly revealed lower numbers of neutrophils within the pulmonary tissues and slightly less numbers in heart, liver, kidney and bowel in animals of the LIM group versus SMC. In addition, we found evidence of improved pulmonary, cardiac, and kidney function in the LIM group as indicated by partially higher svO_2_, and better cardiac output, respectively. Moreover, CK values were lower in the LIM group, however, only after 72 hours. Due to high SEM values at 24 and 48 hours, the interpretation of these data has to be done carefully. Overall, the obtained evidence that posthemorrhagic hemodynamics and metabolism may be better in the LIM group versus SMC should be confirmed by future studies. In addition, the unexpected reduction of monocyte counts by LIM treatment requires further studies.

Although controversial reports exist regarding activation or inhibition of different cell types by Fas stimulation [34] we never observed increased activity upon challenging neutrophils ex vivo with immobilized agonistic Fas. One possible mechanistic explanation of our findings from this in vivo study may be that LIM treatment impairs the motility of circulating neutrophils which may partly result in the failure of neutrophils to transmigrate into tissues. Consequently, the well known neutrophil-mediated disruption of the integrity of endothelial/epithelial layers, impairment of microcirculation, induction of oxidative stress with subsequent lipid peroxidation [35,36] might be limited by LIM. Indeed, neutrophil chemotactic activity has been shown previously to be reduced after LIM treatment [23]. It has been shown previously that blood cells made apoptotic by extracellular exposure to psoralen and UV light exerted anti-inflammatory effects in a graft-versus-host disease model [37]. It would be of interest to find out whether similar anti-inflammatory mechanisms may also exist upon Fas-mediated neutrophil apoptosis. Further evidence that apoptotic cells have anti-inflammatory and immunosuppressive effects when given systemically in a model of murine LPS-induced endotoxic shock has been reported [38].

Herein, shock/resuscitation-induced hemoxygenase-1 (HO-1) expression, probably as a consequence of posthemorrhagic oxidative stress [39,40], was clearly limited in the LIM group in lung, liver, and bowel, organs that frequently are impaired after trauma [41]. HO-1 is known to be induced by oxidative stress and has been shown by others to protect from hemorrhagic shock-induced tissue injury [39]. The finding that gene and protein expression of HO-1 was found to be lower in the LIM group may be a result of limited neutrophil infiltration and neutrophil-mediated oxidative stress.

Shock-induced lipid peroxidation was only observed in the bowels. However, there seems to be no direct correlation between the amount of lipid peroxidation and infiltrated neutrophils within the bowel since only low neutrophil numbers could be detected in the bowel after shock. In contrast, high numbers of apoptotic cells were found in the lamina propria of the bowel in the SMC but not in the LIM group suggesting that inhibition of peripheral inhibition of circulating neutrophils during posthemorrhagic inflammation may result in protection of the bowel. Similarly, shock-induced apoptosis in the lung tissue was also largely prevented by LIM. The underlying mechanisms remain to be defined. One possible explanation might be that LIM protects from the previously described no-reflow phenomenon associated with neutrophils that are sequestered in the capillaries of the tissues, thus damaging the tissue in the absence of overt neutrophil tissue infiltration [41].

## Conclusions

From our data we conclude that targeting of neutrophil Fas during the early posthemorrhagic or posttraumatic time period may ameliorate inflammation-mediated sequelae and thus may be of therapeutic benefit for trauma patients. Due to the small sample size the conclusions have to be made carefully. As usual for explorative studies that have the main objective in the identification of the best primary end point for subsequent confirmative studies, multiple testing of different parameters and time points had to be done, resulting in a reduction of the robustness of the tests performed. Nevertheless, the results obtained provide an interesting basis encouraging further evaluation.

However, the timing of neutrophil inhibition has to be critically considered since inhibition of neutrophil activation might impair anti bacterial phagocytic effects of neutrophils which are essential to prevent sepsis [42,43]. On the other hand, the early prevention of neutrophil-mediated disruption e.g. of the intestinal or pulmonary epithelium might in turn prevent bacterial dissemination and sepsis. Further studies investigating potential clinical benefits of neutrophil Fas-directed immune therapy in patients after hemorrhagic shock or severe trauma are encouraged.

## Competing interests

JA and MS receive salary from and hold shares of LEUKOCARE. None of the other authors have anything to declare.

## Authors' contributions

TL conducted the experiments and draft the manuscript. AP-G, MS, IW, AO, SS, JB, JB-M, AS participated in the experiments including surgical preparation and data collection. WM participated in the histological analysis. AP-G, JA, JW participated in the study design and revised the manuscript critically for important intellectual content. TJ was in charge of he statistical evaluation. MSch conceived of the study, and participated in its design and coordination and draft the manuscript. All authors read and approved the final manuscript.
